# Supplementary material for: Bile Acid Sequestration via Colesevelam Reduces Bile Acid Hydrophobicity and Improves Liver Pathology in Cyp2c70−/− Mice with a Human-like Bile Acid Composition
Source: Biomedicines. 2023 Sep 8;11(9):2495. doi: 10.3390/biomedicines11092495 (PMC10526181; doi:10.3390/biomedicines11092495)

Supplementary Figure 1. Colesevelam does not impact the expression of genes involved in thermogenesis in brown adipose tissue of WTD fed-WT and *Cyp2c70$^{-/-}$* mice.

mRNA levels of genes involved in thermogenesis in brown adipose tissue (BAT) in Cyp2c70$^{-/-}$ mice and WT littermates on control diet and after 3 weeks of Colesevelam treatment. N = 8-9 mice/group. Data are presented as Tukey's box-and-whisker plots. BAT: brown adipose tissue; *Ucp1*: mitochondrial uncoupling protein 1; *Dio2*: iodothyronine deiodinase 2.

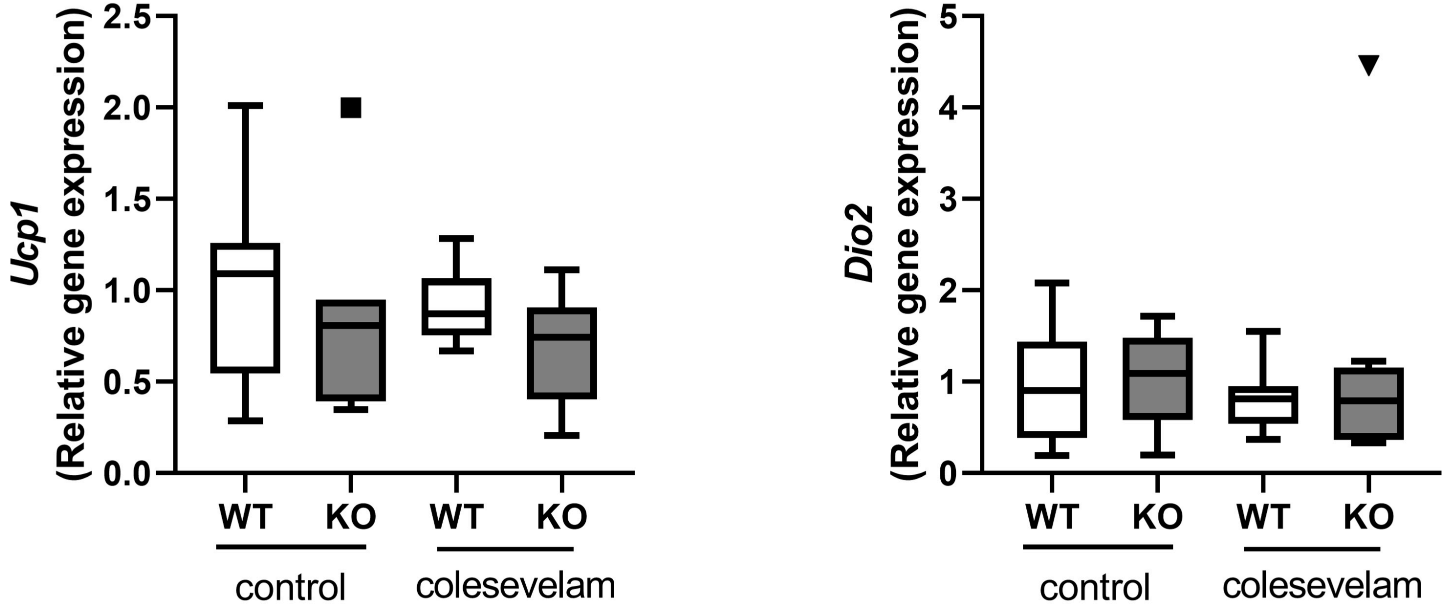

Supplement: Supplementary file 1 [file biomedicines-11-02495-s001.zip › biomedicines-2568501-supplementary.pdf]
